# The Invisible Prevalence of Citizen Science in Global Research: Migratory Birds and Climate Change

**DOI:** 10.1371/journal.pone.0106508

**Published:** 2014-09-03

**Authors:** Caren B. Cooper, Jennifer Shirk, Benjamin Zuckerberg

**Affiliations:** 1 Cornell Lab of Ornithology, Ithaca, New York, United States of America; 2 University of Wisconsin, Madison, Wisconsin, United States of America; University of Colorado, United States of America

## Abstract

Citizen science is a research practice that relies on public contributions of data. The strong recognition of its educational value combined with the need for novel methods to handle subsequent large and complex data sets raises the question: Is citizen science effective at science? A quantitative assessment of the contributions of citizen science for its core purpose – *scientific research* – is lacking. We examined the contribution of citizen science to a review paper by ornithologists in which they formulated ten central claims about the impact of climate change on avian migration. Citizen science was never explicitly mentioned in the review article. For each of the claims, these ornithologists scored their opinions about the amount of research effort invested in each claim and how strongly the claim was supported by evidence. This allowed us to also determine whether their trust in claims was, unwittingly or not, related to the degree to which the claims relied primarily on data generated by citizen scientists. We found that papers based on citizen science constituted between 24 and 77% of the references backing each claim, with no evidence of a mistrust of claims that relied heavily on citizen-science data. We reveal that many of these papers may not easily be recognized as drawing upon volunteer contributions, as the search terms “citizen science” and “volunteer” would have overlooked the majority of the studies that back the ten claims about birds and climate change. Our results suggest that the significance of citizen science to global research, an endeavor that is reliant on long-term information at large spatial scales, might be far greater than is readily perceived. To better understand and track the contributions of citizen science in the future, we urge researchers to use the keyword “citizen science” in papers that draw on efforts of non-professionals.

## Introduction

Citizen science, the practice of involving the public in scientific research, is undergoing a period of rapid growth across numerous disciplines. Effective use of citizen science as a method of data collection relies on techniques from many disciplines, such as science communication, informal science education, and informatics. As an interdisciplinary field of practice, citizen science is experiencing a process of professionalization, which is evident from newly organized societies such as the Citizen Science Association (http://citizenscienceassociation.org), the European Citizen Science Association (http://ecsa.biodiv.naturkundemuseum-berlin.de/), and the Citizen Science Network Australia (http://csna.gaiaresources.com.au). Other hallmarks of professionalization include new educational- and cyber-infrastructure initiatives, international conferences, and numerous advisory boards [1], [2].

Research has documented that citizen science can engage the public through hobbies and games [3], [4], support learning in science, technology, engineering, and math (STEM) [5] provide sources of large and complex datasets that catalyze advances in data visualization [6], [7], advance cyber-infrastructure and new analysis techniques [8], [9], [10], [11], and influence management and policy [12], [13], [14], [15]. But is citizen science effective at science?

Research focused on the scientific value of citizen science has yet to directly quantify the impact of citizen science to any specific area of scientific research, but has instead focused on concerns regarding data quality e.g., [16], [17]. Data from citizen-science efforts can indeed have biases, the largest source of which may originate from the extent to which people self-select to participate, which affect the effort they expend, their level of skill, and the spatiotemporal distribution of the data [18]. Yet, the quality of data collected by volunteers, on a project-by-project basis, has generally been found as reliable as the data collected by professionals in community-based research [19] and contributory projects across a wide variety of subjects, including lady beetles [20], moths [21], wolves [22], trees [23], air pollution [24], light pollution [25], plants [26], pikas [27], invasive plants [28], and bees [29]. Researchers address the biases and variable data quality of volunteered observations through the use of novel online data filters and workflows for detecting erroneous submissions [9], [30], [31], and modified project design and analysis [11], [16], [32]. These advances have been central in the development, vetting, and dissemination of citizen-science datasets that have allowed for new areas of scientific research that would not otherwise be possible; particularly questions requiring data collected over broad spatiotemporal scales.

Many citizen-science programs have operated for decades (some, for more than a century) and frequently span multiple countries and continents [3], [33]. To investigate unanticipated questions arising from global changes, researchers have begun relying on diverse sources of data including the re-purposing of data contributed through citizen science [33]. We expect that research related to climate change may be particularly well suited to draw on citizen science because: (i) the influence of climate change spans broad spatial scales, possibly affecting species throughout their entire ranges, (ii) ecological responses are highly variable across space, that is, not all populations are exposed to similar trends in climate, and (iii) climate-induced impacts occur over long periods of time, from decades to centuries, generally longer than the duration of any scientists' careers or a typical funding cycle.

Given this presumption, we focused on examining citizen science related to research on birds and climate change. Our objectives were to i) quantify the scientific contribution of citizen science to this area of active inquiry, ii) assess whether professionals held volunteer-based research in equal regard to research by professionals, and iii) evaluate the extent to which citizen science was readily visible or noted in the focal studies. Taken together, our goal was to evaluate the use and confidence of citizen science in advancing understanding in an important area of ecology and global change research.

## Materials and Methods

One of the most widely cited lines of evidence that species are responding to modern climate change relates to shifts in phenology. Changing spring phenology in migratory birds is a rapidly developing field of study that has been identified as a critical bellwether for assessing the ecological impacts of climate change. We based our study on a review paper by Knudsen et al. [34] to evaluate the influence of citizen science on the study of climate change research. The Knudsen et al. [34] review represents a critical synthesis of the existing scientific support for the patterns, mechanisms, and consequences of phenological changes in bird migration. The authors (all active researchers in the field of migration phenology) reviewed literature to formulate 10 specific scientific claims about migratory birds and global climate change (Table 1). Also, 18 of the 27 authors scored their opinion regarding the amount of research effort so far invested in each claim (hereafter referred to as “knowledge basis”) and whether each claim held in general (hereafter referred to as “support”), on a continuous scale from 0 (least) to 10 (most) (Table 1). They report the mean for each value and the associated standard deviation.

**Table 1:** Ten claims with mean values for support and knowledge basis from Knudsen et al. [34].

			Expert Opinion			Citizen Science	
	#	Statement	Support	Knowledge basis	# papers*	#	%
Pattern	1	Spring migration advances due to global climate change	8.52	8.28	32	17	53
	2	Phenological response depends on migration distance	5.68	5.99	22	11	50
	3	Climate change affects migratory distance and routes	4.86	3.83	20	15	77
Mechanism	4	Controlling mechanisms are hardwired	4.06	3.79	21	5	24
	5	Changes are mainly due to phenotypic plasticity	4.84	3.79	15	5	33
	6	Phenotypic variability is mainly due to weather *en route*	4.87	4.42	29*	14	48
	7	Responses are constrained by the annual cycle	5.54	3.27	24	11	46
Consequence	8	Increased trophic mismatch on breeding grounds	4.97	4.00	21	5	24
	9	Climate change causes population declines	4.53	3.69	35	20	57
	10	Climate change affects community composition	4.10	2.97	21*	14	67

*excluded papers that we were unable to classify (1 in claim 6 and 1 in claim 10).

The number of papers used for assessment of each claim, and the number and percent of papers that used citizen science.

There were 205 papers referenced in the 10 claims, 15–36 references per claim, in Knudsen et al. [34]. We excluded 42 references that were reviews (including books, chapters, and meta-analyses) from our analysis. We were able to classify 171 of the 173 research papers according to the sources of data: either as including observations collected by volunteers (citizen science) or not (professionals). We noted sources of museum collections (n = 4) and found two of these papers also used data from a citizen science source; we classified the remaining two papers using museum collections as professional. Some (n = 45) papers were referenced in more than one claim, thirteen of which appear in 2–5 claims. We computed the Pearson's correlation between the mean knowledge basis and percent citizen science per claim, and between the mean support and proportion of citizen science per claim. We repeated these analyses using the reported standard deviation associated with knowledge basis and support in relation to proportion of citizen science per claim.

For papers that used citizen-science data, we classified the type of project as: large-scale coordinated scheme (dispersed network of volunteers following a protocol sharing centralized objectives and data management), local volunteers following a protocol but with multiple sampling objectives (e.g., ringers at an observatory), local groups (e.g., local bird clubs and societies), and other (e.g., journals and diaries of amateur naturalists). We also noted the terminology we relied on to classify each citizen-science paper, including explicit mention of volunteers, and/or a specific program, other term, or through contact with the author.

## Results

We examined 173 original research papers that were used by Knudsen and colleagues to formulate 10 central claims about the impacts of climate change on avian migration [34]. We found that 85 of the 171 papers that we could classify were based on citizen science, constituting 5 to 20 papers per claim (Appendix S1). Citizen science heavily informed claims related to ecological patterns and consequences and was less frequently cited for claims about mechanisms (Table 1).

Data from a wide range of citizen-science efforts were included in these papers, including observations from large-scale coordinated programs (n = 35 papers), local volunteers following protocols (n = 40), ad hoc counts from local bird clubs (n = 12 papers), other volunteer sources (n = 4), and some papers (n = 6) drew on several of these citizen-science efforts.

Of the 84 papers that were based on citizen science, 74 were published in, or prior to, 1995, which was when “citizen science” was coined in the context of bird research [35]. Despite the importance of citizen science in substantiating the claims in Knudsen et al. [34], the term “citizen science” *never* appeared in any referenced publications (Appendix S1). The term “volunteer” was used in 37 of the citizen-science papers, typically only in the acknowledgements (Appendix S1). We viewed 37 citizen-science papers as “invisible” because our ability to identify these as citizen science was based on i) the name of specific programs (n = 35; e.g., BTO Common Bird Census), ii) the mention of general programs or efforts (n = 7; e.g., local ornithological societies), iii) terms “bird watchers,” “ringers,” “bander,” “public,” “naturalist,” and “people” (n = 42), iv) by contacting authors and data-providing organizations for confirmation (n = 11 authors contacted and 9 responded), or v) a combination of these identifiers (n = 22). The papers that were difficult to classify were evenly distributed across claims, comprising roughly half of the citizen-science papers per claim.

**Figure 1:**
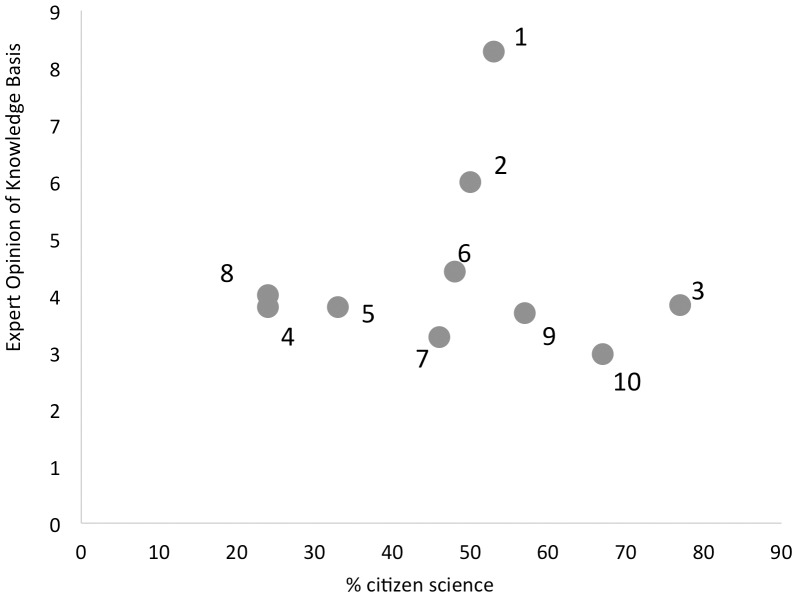
No relationship between the mean values of the expert opinions on the knowledge base of each claim in Knudsen et al. [34] were not correlated to the proportion of citizen science supporting each claim.

Knudsen et al. [34] provided the mean values of the opinions of 18 of their authors on the strength of the knowledge basis and support for each of the claims they reviewed. We found that the mean values of the expert opinions were not correlated to the proportion of citizen science supporting each claim (r = -0.02, *p* = 0.9 for knowledge basis, Figure 1; r = 0.07, *p* = 0.8 for support). Similarly, the standard deviation associated with the mean values of the expert opinions were not correlated to the proportion of citizen science supporting each claim (r = −0.38, *p* = 0.3 for knowledge basis; r = 0.17, *p* = 0.6 for support).

## Discussion

Our findings are strongly indicative of the usefulness and credibility of citizen science in the field of global change research. We found that more than half the central claims about the impacts of climate change on avian migration were based on studies that depended on data from citizen scientists. In addition, we did not find any statistical relationship between the knowledge basis or scientific support for each claim and the proportion of citizen science. The use of citizen science data in an active field of ecological research, such as migration phenology, is strong evidence that any stigma associated with the use of data collected by volunteers is unwarranted. Yet, the contributions of citizen science were not readily detectable in most cases. Thus, the stigma may persist unless researchers begin to draw attention to the citizen-science elements in their research papers.

Citizen science was more critical in supporting claims related to ecological patterns and consequences when compared to claims about mechanisms (Table 1). The reason for this finding highlights a simultaneous strength and limitation of citizen science. One of the primary motivations of developing and deploying a citizen science project is to collect data over spatiotemporal scales that would be difficult (if not impossible) with professional scientists. As a result, these programs have been particularly useful for documenting broad-scale patterns (e.g., macroecology) or long-term consequences (e.g., population trends). Many of these programs, in many ecological disciplines in addition to ornithology, were initiated for purposes other than documenting the ecological responses to climate change, but as our findings emphasize, the scale of data collection has proven essential for analyzing patterns and consequences. Cost savings are another advantage of re-purposing existing data. Climate change studies focused on mechanisms tend to involve experimental investigation (e.g., active or passive warming devices) or more intensive field studies (e.g., use of geolocators). That being said, data generated from long-term citizen science programs have also been repurposed to focus on mechanistic hypotheses and objectives. As an example, several of the papers classified as supporting mechanisms of migration focused on explicit hypotheses such as the geographical variation in phenological mismatch [36], [37] and buffer effects in population dynamics [38]; these hypotheses could not be tested without data from coordinated banding studies involving volunteers in different regions.

Given the invisible prevalence of citizen science in advancing this one area of global change research, we suspect it also common in many other areas of inquiry such as studies of land-use change, invasive species, and environmental pollutants, to name a few. We urge future use of consistent terminology and acknowledgement to facilitate tracking the impact of citizen science across numerous disciplines. Specifically, we urge use of the keyword phrase “citizen science” in papers that rely on scientific contributions from the public. Continued assessment of the value of citizen science in other areas of research could help increase overall public participation as well as identify new frontiers in multiple research fields and improve the interdisciplinary practice of citizen science.

An additional consequence of the invisibility of the scientific impact of citizen science is that projects may miss the broader social impacts of their work. Unique positive societal impacts, such as increased scientific literacy, depend on participants being not merely engaged as instruments or human sensors, but upon being informed and engaged with research progress and outcomes [39]. Yet, many long-running volunteer efforts did not originate with the specific purpose of understanding the consequences of global climate change, and as a result, most of these projects were not designed to foster communication of scientific findings back to project participants; this is particularly true for studies using data from online repositories. Explicit recognition of citizen science in published papers could promote the communication linkages necessary for broader impacts by helping shift public discourse associated with modern climate change from controversy to acceptance. Our findings demonstrate the exceptional value of the efforts of thousands of participants whose data informed the 10 claims, and point to the potential of the millions of global participants whose “invisible” efforts may be contributing to new discoveries.

## Supporting Information

Appendix S1 Research papers in Knudsen et al. [34], classified as involving data from citizen science (yes  = 1, no  = 0).
(XLS)
